# Reduced plasma oxytocin levels in patients with open-angle glaucoma

**DOI:** 10.1007/s10384-025-01248-6

**Published:** 2025-07-14

**Authors:** Yurina Yamada, Kota Sato, Satoru Tsuda, Yu Yokoyama, Noriko Himori, Naoki Kiyota, Naoki Takahashi, Yoko Takeda, Chiaki Yamaguchi, Kazuko Omodaka, Toru Nakazawa

**Affiliations:** 1 Department of Ophthalmology, Tohoku University Graduate School of Medicine, 1-1 Seiryo-machi, Aoba-ku, Sendai, Miyagi Japan 980-8574; 2 Department of Advanced Ophthalmic Medicine, Tohoku University Graduate School of Medicine, Sendai, Miyagi Japan; 3 Department of Ophthalmic Imaging and Information Analytics, Tohoku University Graduate School of Medicine, Sendai, Miyagi Japan; 4 Department of Retinal Disease Control, Tohoku University Graduate School of Medicine, Sendai, Miyagi Japan

**Keywords:** Glaucoma, Oxytocin, Biomarker, Glaucoma progression

## Abstract

**Purpose:**

We explored the role of oxytocin in glaucoma by measuring the blood levels of oxytocin in glaucoma patients, comparing them to normal control subjects, and examining its association with clinical parameters.

**Study design:**

Retrospective cross-sectional study.

**Material and Methods:**

After obtaining informed consent from 181 glaucoma patients and 44 age-matched control participants, we collected blood samples in ethylenediaminetetraacetic acid (EDTA) tubes and centrifuged them at 2000 g for 25 minutes at 4 °C. The resulting plasma was assayed for oxytocin concentration with an Enzyme Linked Immunosorbent Assay (ELISA) kit. We compared oxytocin concentrations in the control and glaucoma groups, and within the glaucoma group, we determined whether the oxytocin levels were correlated with mean deviation (MD) and sectoral total deviation (TD). Furthermore, in 33 patients who underwent at least five visual field tests over a two-year period following oxytocin measurements and received no surgical interventions during that time, we determined whether the oxytocin levels were correlated with MD slope and sectoral TD slopes.

**Results:**

Oxytocin levels in glaucoma patients were significantly lower than in age- and sex-matched normal controls (723.34 ± 303.44 vs. 557.59 ± 296.04 pg/ml, p=0.002). In glaucoma patients, oxytocin levels were significantly correlated with MD and inferior TD after adjustment for age and sex (β=0.149, p=0.041; β=0.156, p=0.034, respectively). There was a weak negative correlation between oxytocin concentration and MD slope (β=-0.334, p=0.084) and a weak negative correlation with central TD slope (β=-0.405, p=0.039), adjusted for age, sex, and history of additional eye drops.

**Conclusion:**

Oxytocin concentrations in glaucoma patients were significantly lower than in normal subjects and associated with the severity and progression of visual field defects. Given the wide variety of the pharmacological actions of oxytocin, it may be involved in the pathogenesis of glaucoma. Our results suggest that plasma oxytocin measurements may open a new avenue for glaucoma care.

**Supplementary Information:**

The online version contains supplementary material available at 10.1007/s10384-025-01248-6.

## Introduction

Glaucoma is the second most common cause of blindness worldwide; it is characterized by retinal ganglion cell (RGC) death and irreversible vision loss [1, 2]. Aging and increased intraocular pressure (IOP) are established risk factors, and lowering IOP is the only evidence-based treatment [3, 4]. In Asia, normal-tension glaucoma (NTG) is the major type of open-angle glaucoma (OAG). IOP-independent factors play a role in the pathogenesis of NTG, so that even with adequate IOP reduction, visual field (VF) defect progression due to glaucoma cannot be prevented in many cases [5–9]. IOP-independent factors include myopia [10, 11], decreased ocular blood flow [12–14], systemic oxidative stress [15, 16], and a low level of Brain-derived neurotrophic factor (BDNF) [17]. Various other IOP-dependent factors may also play a role in the pathogenesis of glaucoma. In other words, in order to prevent blindness due to glaucoma, it is necessary to stratify patients according to various risk factors and establish effective treatment methods.

VF impairment results in a low quality of life and a low level of life satisfaction and happiness because it prevents people from moving around safely and efficiently; eating, dressing, and performing other daily physical activities; and understanding the content of written text, drawings, and pictures [18]. Happiness also has a significant impact on the whole body, leading to systemic disorders such as decreased activity and autonomic imbalance. Dopamine, serotonin, and oxytocin are considered the happy hormones, with oxytocin in particular having an anti-depressant effect [19]. Oxytocin is a peptide hormone consisting of 9 amino acids [20]. It is produced in the paraventricular nucleus and suprachiasmatic nucleus of the hypothalamus, and some oxytocin is transported axonally to the posterior pituitary gland, where it is secreted into the bloodstream. It is also known to play a role in childbirth and lactation. In addition, it is known to be involved in stress reduction [21], affection [22], and social behavior [23, 24]. It also influences factors such as trust, tolerance, and emotional understanding. Oxytocin receptors are expressed in a variety of locations [25] and are reported to play different roles in various neurotransmitters and sites [26]. Notably, oxytocin is localized in the cone photoreceptors, and oxytocin receptors are expressed in the retinal pigment epithelium (RPE) in rhesus monkeys, suggesting that oxytocin contributes to signaling in the outer retina [27]. However, there is an unclear relationship between oxytocin and visual loss.

In this study, we focused on the various roles of oxytocin in glaucoma, including its sociological and neuroscientific aspects. To date, there have been no investigations that measured blood oxytocin levels in glaucoma patients. In this study, we attempted to establish a method to measure blood oxytocin levels in glaucoma patients and compare the results with those from normal subjects, as well as oxytocin levels with various measurement parameters of glaucoma, including VF defect progression, to investigate the role of oxytocin in glaucoma and open a new avenue of research.

## Materials and methods

### Patient criteria

In accordance with the principles of the Declaration of Helsinki, informed consent was obtained prior to participation in this study and approval was obtained from the institutional review board of Tohoku University Graduate School of Medicine (No. 2023-1-748).

This study recruited patients who visited Tohoku University Hospital from November 2015 to March 2022 and were diagnosed with glaucoma by a glaucoma specialist (T.N.). Glaucoma was defined in this study as the presence of an abnormal glaucomatous optic disc (with diffuse or focal thinning of the neuroretinal rim) and corresponding glaucomatous VF defects, defined by the Anderson-Patella criteria as the presence of one or more of the following: (1) a cluster of three points with reduced sensitivity at a probability of <5% on the pattern deviation map in at least one hemifield (including ≥1 point at a probability of <1% or a cluster of two points at a probability of <1%), (2) glaucomatous hemifield test results outside the normal limits, and (3) a pattern standard deviation beyond 95% of normal limits, as confirmed in at least two reliable examinations. NTG was diagnosed if peak IOP was 21 mmHg or less without any glaucoma medication, and primary OAG (POAG) was diagnosed if peak IOP was more than 21 mmHg. Eyes with cataract (i.e., a lens nucleus with a grade of 3 or more in the Emery-Little classification, posterior subcapsular cataracts, or cortical cataracts), any ocular surface disease, any corneal disease, any vitreoretinal or optic nerve diseases other than glaucoma, or a history of intraocular surgery other than cataract surgery were excluded from the current study. A total of 181 eyes of 181 OAG patients diagnosed with POAG and NTG were included. We examined the correlation between oxytocin levels and MD slopes as well as superior, central, and inferior TD slopes. MD slope and sectoral TD slope were calculated using the date of oxytocin measurement as the baseline and were based on at least five Humphrey 24-2 VF tests performed over a period of at least two years. The analysis included 33 OAG patients who did not undergo any surgical interventions, including interventions for cataract or glaucoma, during the observation period.

The normal control group included 44 eyes of 44 patients who underwent ophthalmologic examinations at health checkups in Taiwa, Miyagi prefecture, on May 26 and 29 or June 3 and 4, 2017. Details were previously reported by Sato et al. [28]. The definition of a normal subject was: no abnormalities in the anterior segment or intermediate translucent bodies in a slit lamp examination, visual acuity of 0.7 or better, no abnormalities in fundus photographs or optical coherence tomography (OCT) as judged by an ophthalmologist, no abnormalities in Humphrey frequency doubling technology (FDT) screening, HbA1c less than 7.0%, and systolic blood pressure less than 160 mmHg.

### Methods of examination

Ophthalmic examination equipment was used to measure central corneal thickness (CASIA2; Tomey Corporation) and ocular axis length (OA2000; Tomey). Corneal angle was confirmed in GS-1 (Nidek Co., Ltd) images. The VF was measured with the Swedish interactive thresholding algorithm–standard Humphrey Field Analyzer 24-2 program (Carl Zeiss Meditec). For the MD slope and sectoral TD slope analyses, only measurements with fixation errors <20%, false positives <33%, and false negatives <33% were used. The site-specific VF was evaluated with reference to the VF sector map established by Garway-Heath et al. [29]. Averaged superior, central, and inferior TD was calculated and used for the statistical analysis. Circumpapillary retinal nerve fiber layer thickness (cpRNFLT) was measured with DRI-Triton (Topcon).

### Oxytocin measurement

Blood collected in ethylenediaminetetraacetic acid (EDTA) tubes was immediately stored at 4 °C. Plasma was prepared by centrifugation (2000 g, 25 min, 4 °C) within 2 hours of collection. The resulting plasma was frozen at − 80 °C immediately after centrifugation until the assay was performed. Thawed samples were assayed for oxytocin concentration using the ELISA kit (Fujifilm Wako Pure Chemicals) according to the manual.

### Statistical analysis

For the analysis, the Mann-Whitney U test was used to compare age and IOP in normal subjects and in patients with glaucoma, and the chi-square test was used to compare the male to female ratio. The comparison of oxytocin levels and various measurement parameters in the glaucoma group was conducted using a linear mixed-effects model. The Mann-Whitney U test was used to determine the correlation between oxytocin concentrations and various types of glaucoma eye drops in the glaucoma group. The significance level for the above analysis was set at 5%. All statistical analyses were performed with R software version 4.3.0 (available at https://www.R-project.org/).

## Results

The backgrounds of the normal subjects and glaucoma patients who participated in this study are summarized in Table 1. There was no significant difference in age between normal subjects (66.57 ± 11.14 years) and glaucoma patients (67.14 ± 7.62 years; p=0.866). The male-to-female ratio was 19:25 in the normal group and 92:89 in the glaucoma group, with no significant difference (p=0.214). Similarly, IOP did not significantly differ between the two groups (13.36 ± 2.19 mmHg vs. 13.59 ± 4.32 mmHg; p=0.724). In the glaucoma patients, VF parameters were as follows: MD − 14.63 ± 7.42, central TD − 13.13 ± 9.29, superior TD − 15.48 ± 9.93, and inferior TD − 14.62 ± 9.91.

**Table 1:**
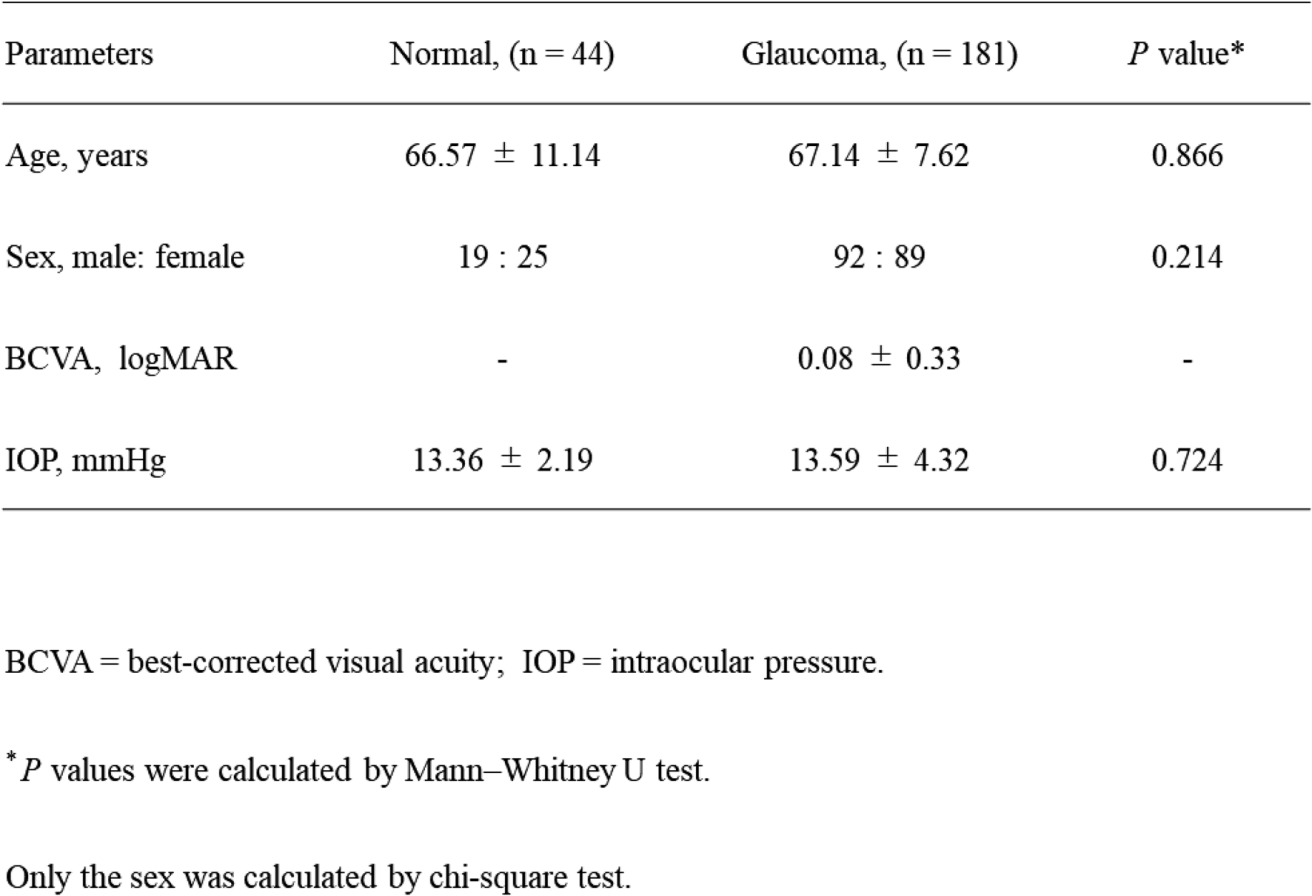
Clinical background factors in normal and glaucoma

**Fig. 1:**
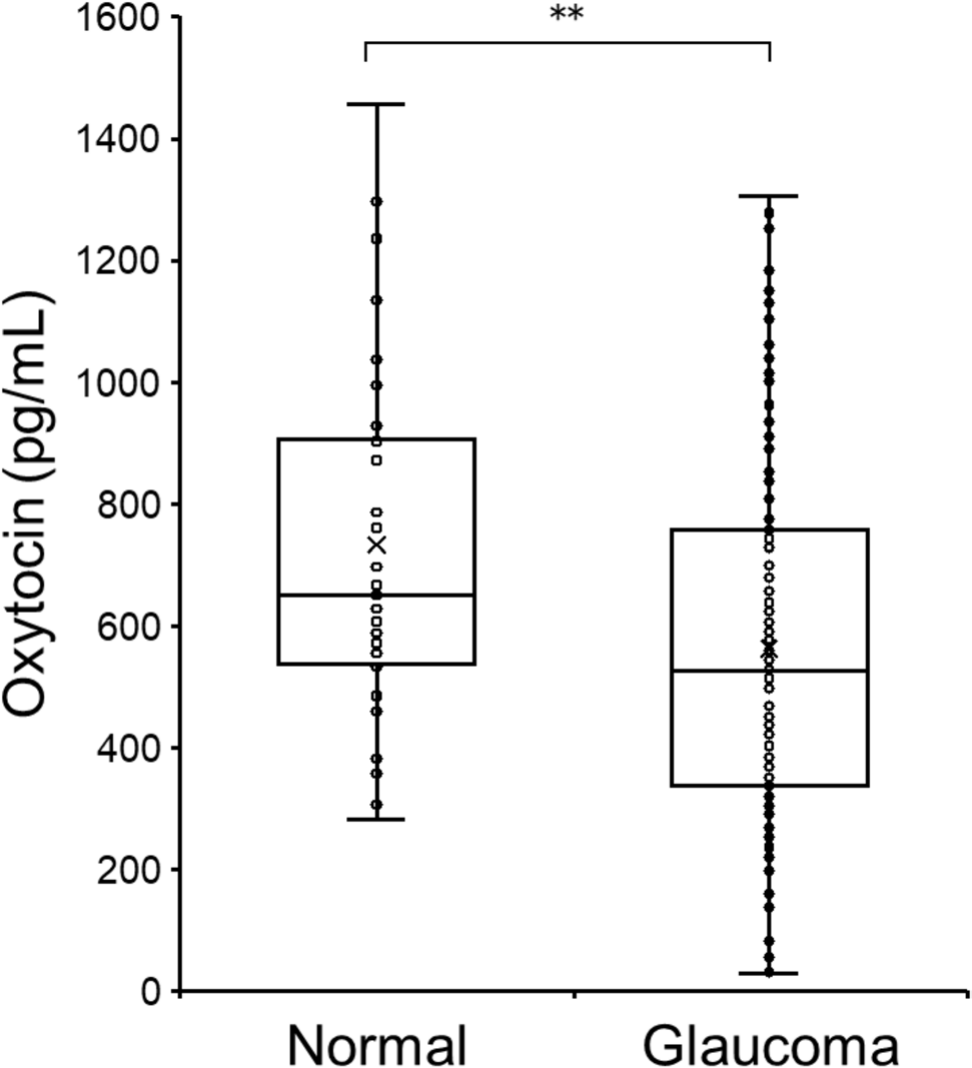
Comparison of plasma oxytocin levels in normal controls and glaucoma patients. The box-and-whisker diagrams shows oxytocin concentrations. The Whitney U test was used to compare the two groups. **P<0.01.

Fig. 1 shows a comparison of plasma oxytocin levels in the glaucoma patients and normal subjects. Oxytocin levels in the normal controls were significantly higher than in the glaucoma patients (723.34 ± 303.44 vs. 557.59 ± 296.04 pg/ml, p=0.002).

**Fig. 2:**
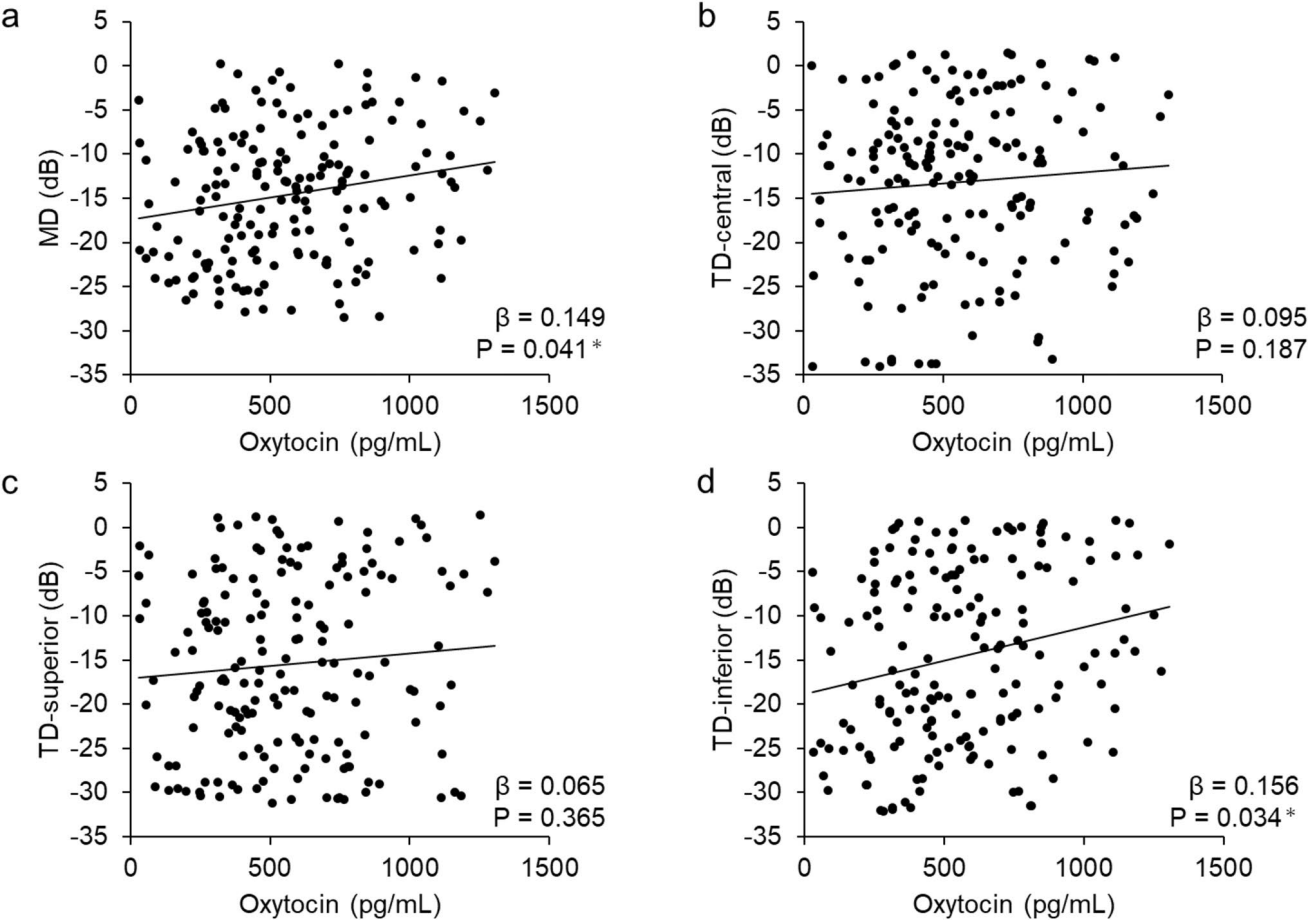
Association between oxytocin levels and mean deviation (MD) and total deviation (TD) in the superior, central, and inferior sectors in glaucoma patients, adjusted for age and sex. **a**: MD (β=0.149, p=0.041). **b**: Central TD (β=0.095, p=0.187). **c**: Superior TD (β=0.065, p=0.365). **d**: Inferior TD (β=0.156, p=0.034)

Table 2 and Fig. 2 present the associations between oxytocin levels and MD or sectoral TD in the glaucoma patients, as analyzed using a linear mixed-effects model and illustrated by scatter plots. In the single correlation analysis, age was significantly associated with oxytocin concentration (r=− 0.278, p=0.001). The male to female ratio was significantly associated with oxytocin concentration (r=0.152, p=0.023). On the other hand, there were no significant differences in visual acuity, IOP, central corneal thickness, axial length, or cpRNFLT (p>0.05). Oxytocin levels were significantly correlated with MD and inferior TD (β=0.149, p=0.041 [Fig. 2a] and β=0.156, p=0.034 [Fig. 2d], respectively), after adjustment for age and sex.

**Table 2:**
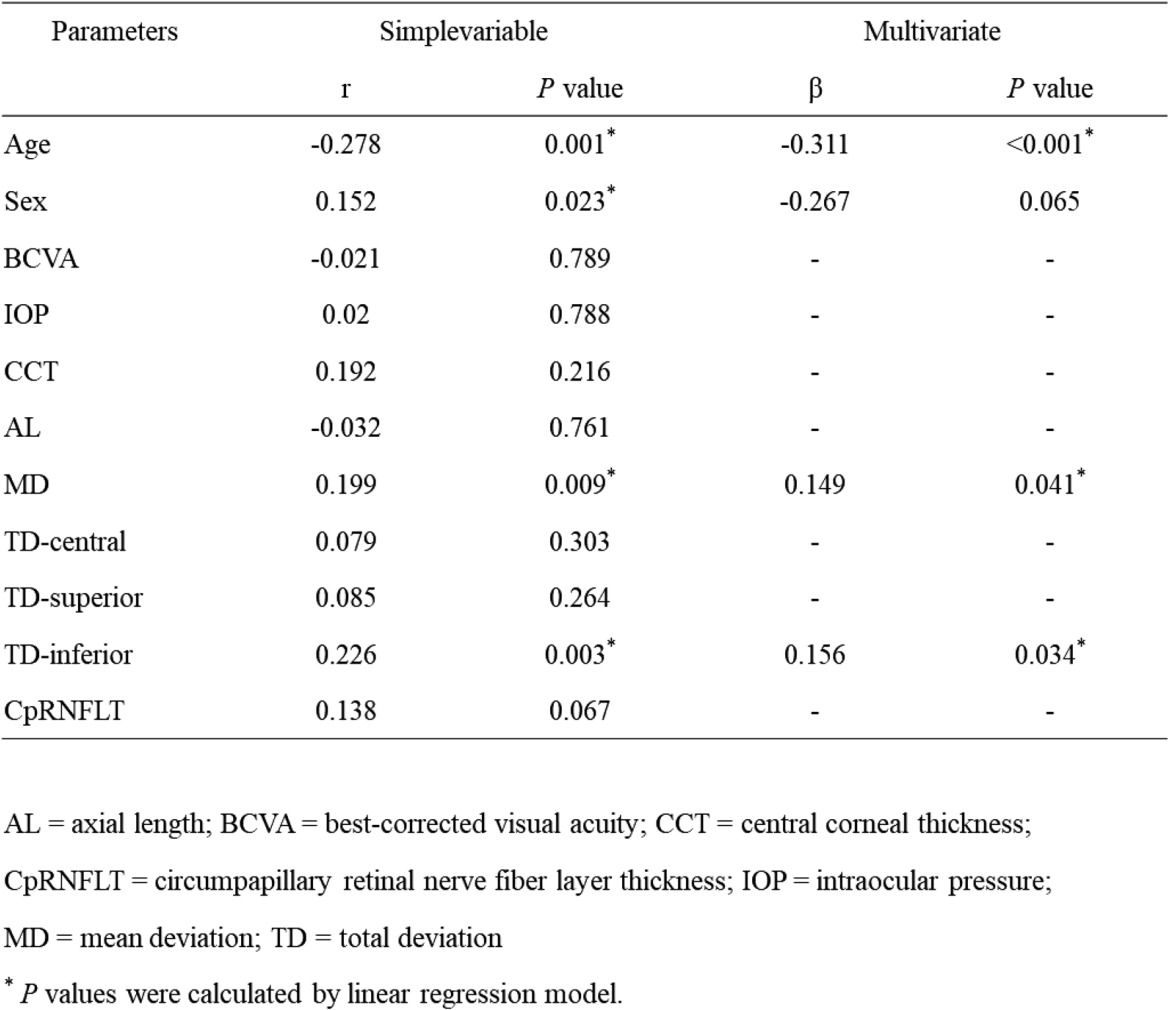
Relationship between oxytocin levels and parameters in glaucoma

**Fig. 3:**
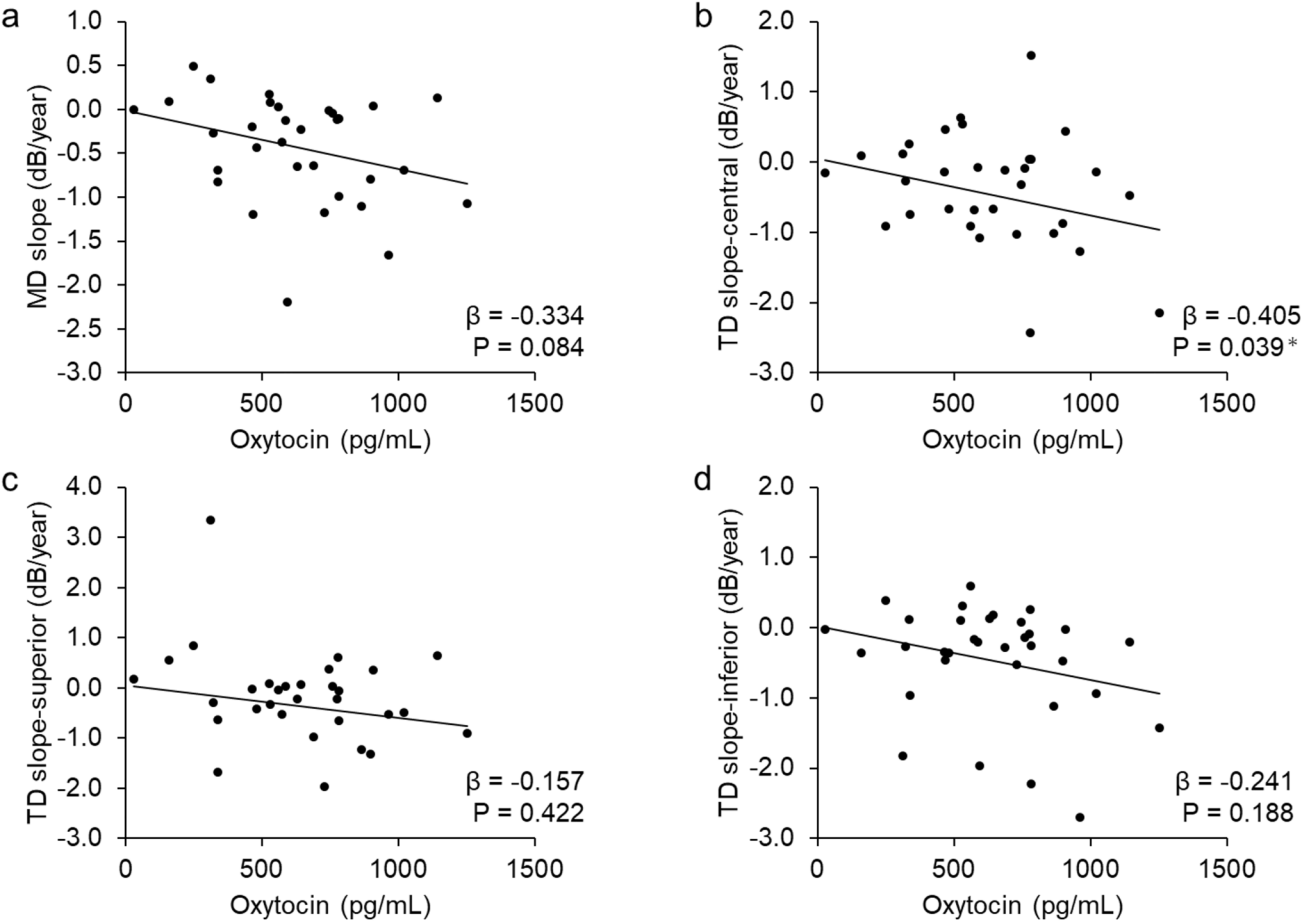
Association between oxytocin levels and mean deviation (MD) slope and total deviation (TD) slope in the superior, central, and inferior sectors, adjusted for age, sex, and additional eye drop use. **a**: A trend toward a negative correlation was observed between MD slope and oxytocin level (β=-0.334, p=0.084). **b**: Central TD slope showed a weak negative correlation (β=-0.405, p=0.039). **c**: Superior TD slope showed no significant correlation (β=-0.157, p=0.422). **d**: Inferior TD slope showed no significant correlation (β=-0.241, p=0.188).

Fig. 3 shows the associations with oxytocin concentrations for MD slope and sectoral TD slopes. For this analysis of visual field progression, patient data (n=33) are presented in Supplementary Table 1. There was a negative correlation between oxytocin levels and MD slope (β=− 0.334, p=0.084; Fig. 3a) and a weak negative correlation with central TD slope (β=− 0.405, p=0.039; Fig. 3b), after adjustment for age, sex, and history of additional eye drops. In contrast, no significant correlations were found between oxytocin levels and superior and inferior TD slopes (β=− 0.157, p=0.422 [Fig. 3c] and β=− 0.241, p=0.188 [Fig. 3d], respectively).

Table 3 shows the type, number, and percentage of glaucoma patients receiving glaucoma eye drops. The average duration of eye drop use among all patients was 15.77 ± 6.55 years, which was not associated with oxytocin levels (r=− 0.234, p=0.491). Prostaglandins were the most commonly used eye drops, used by 92.9% of the participants, followed by β-blockers (72.5%), carbonic anhydrase inhibitors (69.8%), α-2 stimulators (54.9%), rho kinase inhibitors (31.3%), α-1 inhibitors (5.5%), pilocarpine hydrochloride (4.4%), and selective EP2 receptor agonists (0.6%). There were no significant differences in oxytocin levels between users and non-users for any of these medications (p>0.05).

**Table 3:**
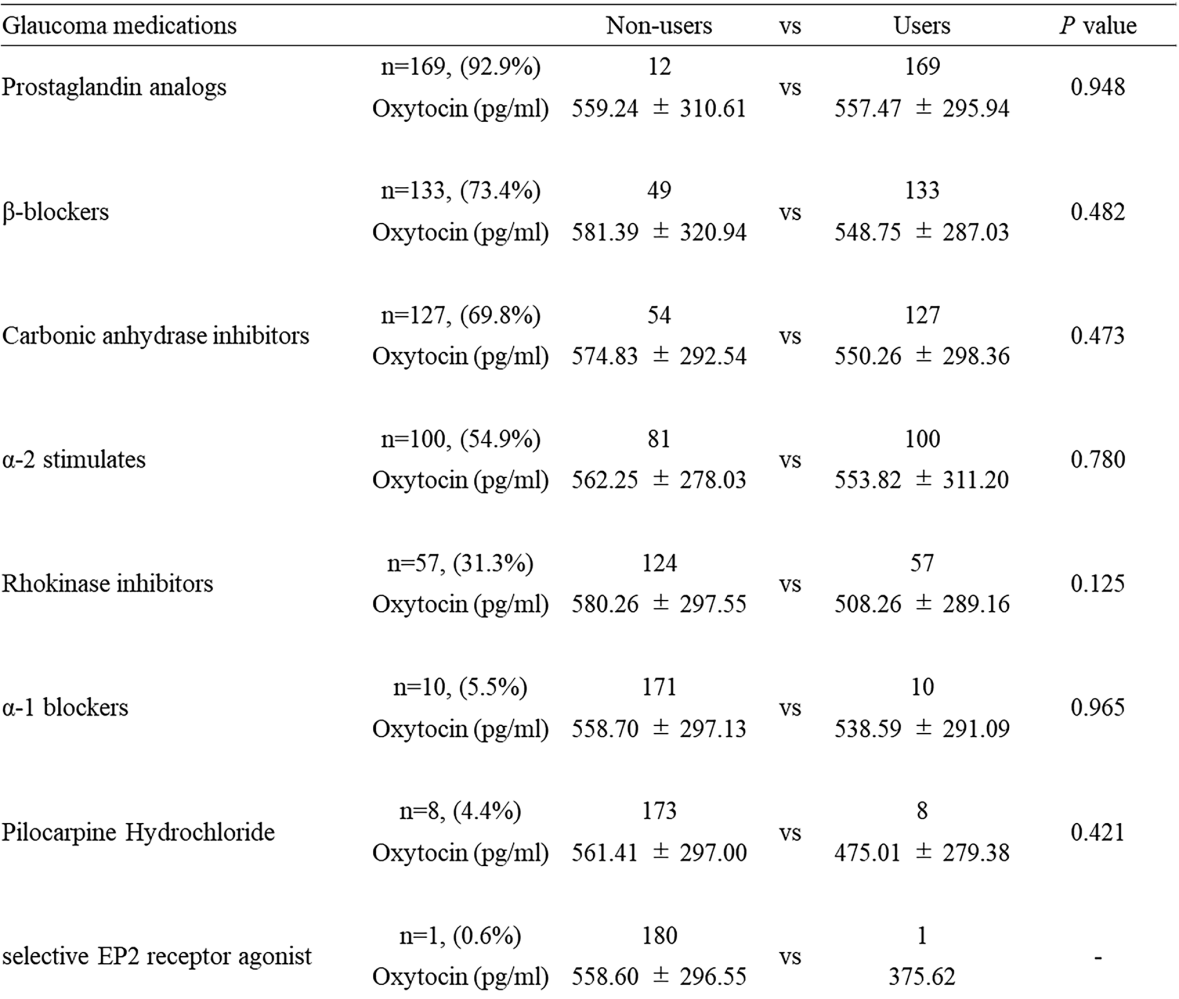
Oxytocin concentration for each glaucoma medications (eye drop)

## Discussion

The present study found that oxytocin concentrations in the blood of glaucoma patients were significantly lower than in age- and sex-matched normal subjects. In glaucoma patients, after adjusting for age and sex, oxytocin concentration was still significantly associated with MD and inferior TD. In addition, there was a negative correlation between oxytocin levels and MD slope and a weak negative correlation specifically with central TD slope, after adjustment for age, sex, and history of additional eye drops. In the participants with glaucoma, oxytocin concentration was not affected by the type or number of ophthalmologic medications. Oxytocin is reported to be involved in the homeostatic balance of the autonomic nervous system [30], and its association with autonomic imbalance has also been previously noted in glaucoma [31]. The above results indicate that oxytocin levels are correlated with the severity of glaucoma, making this a hormone that deserves further attention and opening a window of opportunity for further research.

In the present study, we confirmed that oxytocin levels are low in glaucoma patients. There are few reports on the relationship between glaucoma and oxytocin. However, it has recently been demonstrated that oxytocin secretion is promoted by light stimulation via melanopsin, a photosensitive protein expressed in intrinsically photosensitive RGCs (ipRGCs) that regulate circadian biological rhythms; furthermore, oxytocin has been shown to play an important role in brain synaptogenesis during development [32]. It is also reported that melanopsin-positive ipRGCs are decreased in glaucoma patients, and that melanopsin decreases with the progression of glaucoma [33]. A normal circadian rhythm is necessary to regulate IOP, and sleep disturbances are more prevalent in patients with glaucoma [34, 35]. In addition, melatonin is reported to decrease with the progression of glaucoma, suggesting a circadian disruption such as the absence of nocturnal melatonin suppression [36]. These findings show that circadian rhythm disruption can promote glaucoma pathogenesis [37]. Moreover, oxytocin reduction may disturb the circadian rhythm and reflect the decrease in melanopsin-positive ipRGCs in glaucoma.

As mentioned in the previous paragraph, a decrease in ipRGCs may decrease oxytocin secretion, but oxytocin was originally known to be associated with well-being; therefore, it is also known as a happy hormone. Visual impairment due to glaucoma leads to loss of mobility and difficulty in reading, and has a significant impact on social isolation and negative emotions [18, 38]. Questionnaire assessments related to quality of life in glaucoma patients show that the more severe the case of glaucoma, the lower the psychological health score of the individual [39]. This suggests that glaucoma patients have reduced well-being and their reduced positive feelings may be reflected in their oxytocin level.

Oxytocin is also involved in autonomic regulation. We have previously reported an association between glaucoma and the autonomic nervous system. Although many reports suggest that people with a predominant sympathetic nervous system are more prone to glaucoma, our data show that individuals with a particularly weak sympathetic response to orthostatic load had more severe VF damage, particularly in the inferior VF [40]. Impairment of the inferior VF is also associated with ocular blood flow disturbances and sleep apnea syndrome [41]. This study shows an association between oxytocin levels and MD and inferior VF impairment, which may be influenced by oxytocin as well as blood flow and sleep apnea syndrome. Furthermore, this study demonstrates that oxytocin levels are associated with VF defect progression, particularly in the central field, suggesting a possible link between well-being and glaucoma progression. It is expected that glaucoma and VF impairment may lower the quality of life and influence the sense of well-being. Thus, since glaucoma is related to abnormal autonomic balance, our findings suggest that a low oxytocin level is associated with glaucoma.

Systemic side effects following topical administration of ophthalmic solutions occur primarily as a result of drug absorption through the nasopharyngeal mucosa after passing through the nasolacrimal duct. β-blockers are known to have adverse cardiovascular effects, inducing, for example, bronchospasm, bradycardia [42], and, less frequently, depression [43]. Various eye drops are known to affect the autonomic nervous system, including α1-blockers. In the present study, the level of oxytocin was not affected by the type of eye drops, including prostaglandin analogs (Table 3). Previous work reports that PGF2α stimulates oxytocin release in human luteal cells [44]. The PGF2α receptor has been shown to become desensitized after one hour of stimulation in infusion experiments based on the sheep corpus luteum [45]. This suggests that prostaglandin-analog eye drops may promote oxytocin release, but that this may be a transient effect caused by the desensitization of the PGF2α receptor, with the result that there is no change in oxytocin levels in glaucoma patients treated with prostaglandin analogs. Interestingly, PGF2α stimulates the expression of oxytocin receptors [46]. Although there is no change in the oxytocin levels in patients treated with prostaglandin analogs, oxytocin signaling may be activated. Systemic drugs also affect the autonomic nervous system, although we observed no significant differences among the many drug categories. Overall, the oxytocin reduction we found in the present study was drug independent in glaucoma patients and may have been due to visual field impairment and mental changes.

This study has several limitations: (1) Information on sleep duration and whether the glaucoma patients were taking sleep-inducing drugs is missing. Therefore, it was not possible to verify the association between the circadian rhythm and oxytocin levels in the glaucoma patients included in this study. (2) IOP was not measured in a way that accounted for diurnal variation. As a result, the relationship between oxytocin concentrations and diurnal IOP fluctuations remains unclear and requires further investigation. (3) No association was found between oxytocin levels and the use or duration of glaucoma medications. However, as this study was conducted in a single university hospital and included many patients with long-term treatment histories of varying duration and severity, the impact of topical medications on oxytocin levels remains uncertain. In addition to these limitations, this study was cross-sectional, so the causal relationship remains unclear, even though the number of cases was enough to show a difference between participants with and without OAG. We are looking forward to performing a future prospective study to determine whether the concentration of oxytocin precedes or follows the presence of glaucoma.

In conclusion, we found that oxytocin levels in glaucoma patients were significantly lower than in normal subjects, suggesting that glaucoma patients may have lower well-being. This suggests that measuring blood oxytocin levels may also be useful in assessing the well-being of glaucoma patients.

## Supplementary Information

Below is the link to the electronic supplementary material.

Supplementary file1 (PDF 43 KB)
