# Supplementary material for: Reduced plasma oxytocin levels in patients with open-angle glaucoma
Source: Jpn J Ophthalmol. 2025 Jul 14;70(1):190–8. doi: 10.1007/s10384-025-01248-6 (PMC12948832; doi:10.1007/s10384-025-01248-6)
Supplement: Supplementary file 1 — Supplementary file1 (PDF 43 KB) [file 10384_2025_1248_MOESM1_ESM.pdf]

| Characteristic | n = 33 |
|---|---|
| Age, years | 62.24 ± 4.34 |
| Sex, male: female | 13 : 20 |
| Additional eye drops, n (%) | 10, (30.3%) |
| Oxytocin, pg/ml | 633.78 ± 277.02 |
| MD, dB | -9.75 ± 5.49 |
| TD-central, dB | -9.33 ± 7.60 |
| TD-superior, dB | -11.88 ± 10.09 |
| TD-inferior, dB | -8.34 ± 7.58 |
| MD slope, dB/year | -0.43 ± 0.60 |
| TD-slope central, dB/year | -0.46 ± 0.90 |
| TD-slope superior, dB/year | -0.35 ± 1.15 |
| TD-slope inferior, dB/year | -0.46 ± 0.79 |
